# Visceral Leishmaniasis With Blastocystis Co-infection: A Case Report

**DOI:** 10.7759/cureus.44050

**Published:** 2023-08-24

**Authors:** Tengfei Wang, Debby Rampisela

**Affiliations:** 1 Pathology, Baylor Scott & White Health, Temple, USA

**Keywords:** blastocystis, visceral leishmaniasis, splenomegaly, pancytopenia, diarrhea

## Abstract

Visceral leishmaniasis (VL) is a form of leishmaniasis, which causes significant mortality if untreated. The coexistence of VL with *Blastocystis* infection has not been well-documented in the literature. In this paper, we present the case of a 72-year-old male who experienced four months of recurrent diarrhea and later developed weight loss, fever, night sweats, and pancytopenia. The stool ova and parasite (O&P) examination revealed *Blastocystis* spp. vacuolar bodies and he was treated with metronidazole which resolved the diarrhea but not other symptoms. Further evaluation, including an abdominal Computed Tomogram (CT) scan and ultrasonography (USG), revealed splenomegaly. A splenic biopsy confirmed VL with numerous *Leishmania* amastigotes. Treatment with Amphotericin B led to clinical improvement. This paper discusses the clinical and diagnostic features of VL and *Blastocysti*s, highlighting their differential diagnosis, and available treatments.

## Introduction

Leishmaniasis affects 15 million people in 83 countries, with 1.5-2 million new cases annually, predominantly affecting populations in poverty [1]. It is a vector-borne disease transmitted by phlebotomine female sand flies. *Leishmania* infects around 70 animal species, including humans. Over 20 species of the genus *Leishmania* have been identified to infect humans [2].

Leishmaniasis exhibits various clinical manifestations, including visceral form (VL, also known as kala-azar), cutaneous form (CL), and mucocutaneous/mucosal (ML) form. Particularly, VL accounts for 50,000-90,000 new cases per year, has a high mortality rate if untreated, and is prevalent in specific countries, namely Bangladesh, Brazil, Ethiopia, India, South Sudan, and Sudan [2]. Although not considered endemic in the United States among humans, traveler-related VL cases occur in the US [3]. Co-infections with other diseases, such as HIV, tuberculosis, helminthiasis, malaria, and leprosy, are common in endemic regions, leading to severe outcomes and highlighting the need for accurate diagnosis [2].

*Blastocystis hominis* was previously used to describe *Blastocystis* organisms found in humans. However, the preferred scientific term now is *Blastocystis* spp. to indicate the presence of "multiple species" due to genetic diversity. *Blastocystis* infection is commonly referred to as blastocystosis. The prevalence of *Blastocystis* spp. varies significantly worldwide, ranging from 22% to 100%, commonly influenced by factors such as aging, low socioeconomic condition, contaminated drinking water, poor hygiene, and travel [4].

## Case presentation

**Figure 1:**
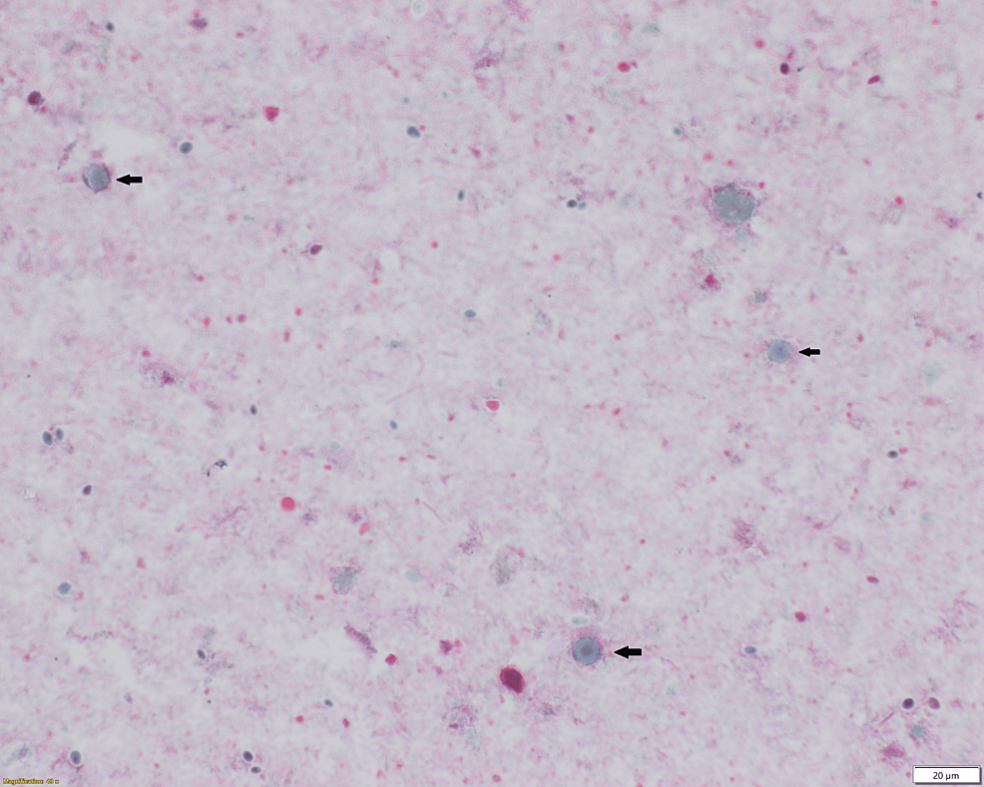
Stool O&P test results Stool ova and parasite (O&P) test results show the vacuolar form of *Blastocystis* spp. (black arrows), Wheatley’s modified Trichrome (400 X).

A 72-year-old male traveler from Venezuela presented with four months of recurrent watery, and at times, foul-smelling diarrhea. After approximately three months of recurring diarrhea, he began experiencing an occasional low-grade fever, reaching up to 100.3°F, along with night sweats, loss of appetite, and generalized weakness. Additionally, he had lost approximately 20 kg in weight over the past few months. The patient had a history of type 2 diabetes, hypertension, and uneventfully chikungunya and Zika virus infection in the past. He had contact with cattle/livestock and chickens and had no history of mosquito bites. His complete blood count (CBC) showed pancytopenia with WBC 2.3×10^9^/L (reference range: 4.8-10.8×10^9^/L), RBC 4.02×10^12^/L (reference range: 4.70-6.10×10^12^/L) and platelet count 69×10^9^/L (reference range: 150-450×10^9^/L). His stool O&P was positive for vacuolar form of *Blastocystis* spp. (Figure 1) and he was treated with metronidazole.

**Figure 2:**
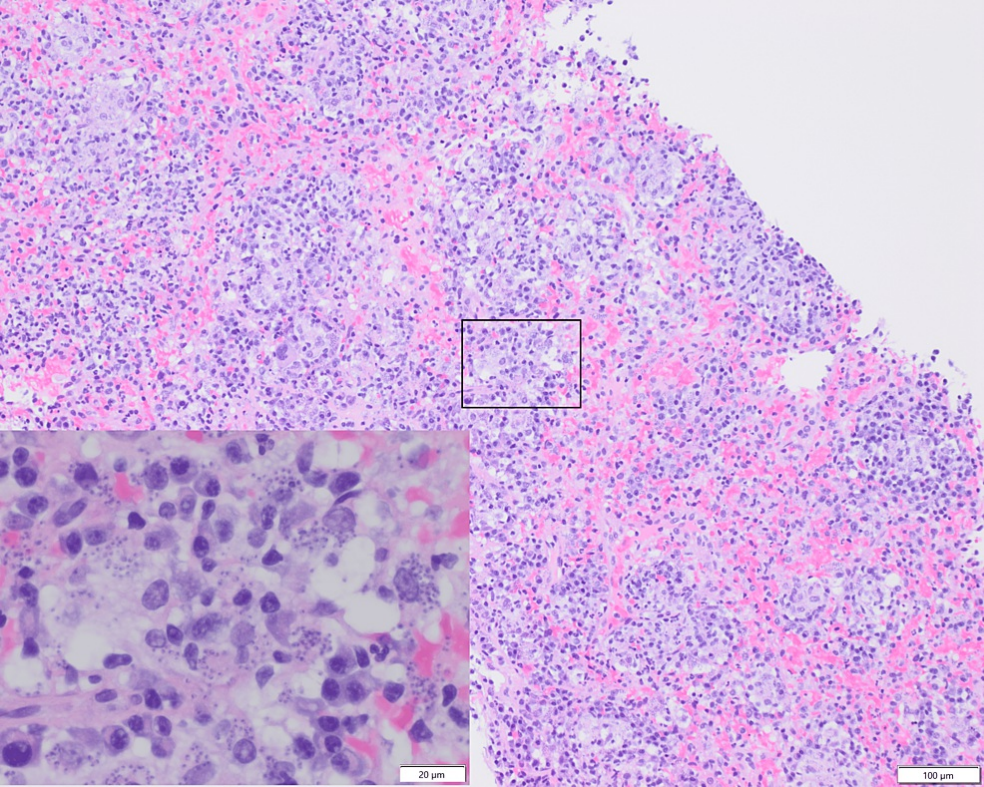
Spleen core biopsy results Spleen core biopsy shows granulomatous chronic inflammation with numerous *Leishmania *amastigotes within the macrophages (H&E stain, 100X, inset 400X).

**Figure 3:**
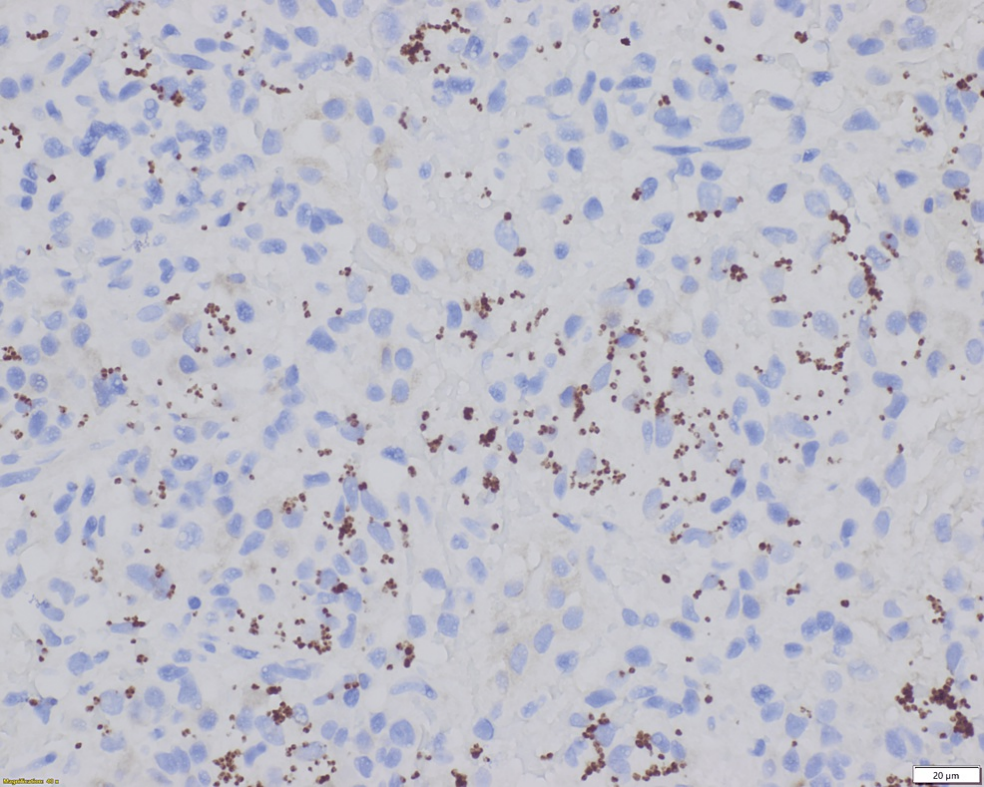
Spleen core biopsy results CD1a immunostain highlights the *Leishmania *amastigotes within the macrophages (400X).

His diarrhea resolved; however, other symptoms persisted. Due to the persistent symptoms and pancytopenia further workup to rule out hematolymphoid malignancy was done. Abdominal CT scan and USG showed splenomegaly without focal mass, measuring up to 19.5 cm. Bone marrow aspiration with flow cytometry, and peripheral blood with flow cytometry showed no significant findings. He then underwent a splenic core biopsy. The biopsy showed splenic tissue with granulomatous chronic inflammation with numerous organisms (Figure 2). The organisms were positive for CD1a (Figure 3), and negative for gram, Periodic acid-Schiff (PAS), Fite, and Grocott-Gomori's Methenamine Silver (GMS) stains, supporting *Leishmania *amastigotes.

A review of the cytospin slide from the spleen also showed numerous *Leishmania *amastigotes. The flow cytometry of the spleen showed no significant findings. HIV, Arbovirus panel (dengue, chikungunya and Zika virus) and TB testing were all negative. Further review of the history revealed that he lived in the city in Venezuela, where there had been reported cases of leishmaniasis. The patient was hospitalized and treated for VL with an infusion of liposomal amphotericin B daily × 5 days and then on days 14, 21. He tolerated the treatment well and was clinically improved after the treatment. He is currently under clinical monitoring and observation for improvement or relapse.

## Discussion

We reported a unique case of VL with* Blastocystis *co-occurrence. The patient presented with recurrent diarrhea, followed by fever, weight loss, and night sweats. Given that diarrhea was the main initial complaint and considering the patient's history of animal contact, the primary clinical differential diagnosis was gastrointestinal tract infection. Stool examination revealed the presence of *Blastocystis* vacuolar form, leading to treatment with metronidazole. However, the patient’s other symptoms persisted, and the presence of pancytopenia raised concerns about potential malignancies commonly seen in the elderly, such as lymphoma and leukemia. Subsequent abdominal imaging and eventually spleen biopsy/cytospin confirmed the diagnosis of VL.

VL is primarily a poverty-related disease prevalent in Africa, Asia, and Latin America. It is associated with malnutrition, population migration, poor housing conditions, weakened immune systems, and lack of resources. VL transmission varies by geographic region; for example, *Leishmania donovani* is transmitted by the female sand fly *Phlebotomus argentipes*, with humans as the sole reservoir host in the Indian subcontinent and East Africa. In contrast, *Leishmania chagasi*, also known as *Leishmania infantum*, is transmitted by *Lutzomyia longipalpis*, with humans and dogs as reservoirs in Europe, North Africa, and Latin America [5]. Rarely, transmission can also occur via blood transfusions, needle sharing, from mother to fetus, or organ transplantation [1]. Travel-related VL cases have been reported in the United States among humans.

VL is an infection that encompasses a range of disease manifestations, varying from asymptomatic to symptomatic. Asymptomatic VL cases outnumber symptomatic ones, with rates 5-10 times higher [6]. Symptomatic VL presents with fever, cold sweats, weight loss, malaise, hepatosplenomegaly, and pancytopenia [7]. Diarrhea can also occur in conjunction with other symptoms [8], but chronic diarrhea is rare [9]. Symptomatic disease is more common in children (< 10 y/o) and immunocompromised populations [10]. 

Visualization of amastigotes in tissue is the gold standard for diagnosing VL in humans. Lymph nodes, bone marrow, and spleen samples are commonly tested, with sensitivity above 90% for spleen [7]. Using light microscopy with stains like hematoxylin and eosin (H&E), Giemsa or Wright-Giemsa, amastigotes can be observed within macrophages and extracellularly as dot-like structures (400X). Amastigotes are non-flagellated oval to round bodies measuring 1-4 μm with a rod-shaped kinetoplast and a circular nucleus (1000X) [11]. Immunohistochemistry with specific antibodies such as monoclonal anti-Leishmania antibody (G2D10) and anti-CD1a antibody, clone MTB1, can also be helpful in the diagnosis. Special stains like GMS, PAS, and Fite stains are negative for amastigotes [12-15].

In routine H&E stain tissue, the main morphologic differential diagnosis for *Leishmania* amastigotes includes* Trypanosoma cruzi* and *Histoplasma capsulatum*. *Trypanosoma cruzi* amastigotes share a similar size (2-6 µm) with a single nucleus and kinetoplast but are localized in cardiac muscles, not macrophages. On the other hand, *Histoplasma capsulatum* also has a comparable size (2-4 μm) but can be identified by using GMS or PAS stains, revealing oval, narrow-based budding yeasts. Distinguishing these organisms in routine H&E stain on tissue slides can be challenging [7].

Other diagnostic tests for leishmaniasis include PCR tests, immunoassays such as enzyme-linked immunosorbent assay (ELISA), western blot, the indirect fluorescent antibody test (IFAT), immunofluorescence, and the indirect hemagglutination assay (IHA); and agglutination tests, such as the direct agglutination test (DAT) [10]. 

Chemotherapy is the primary treatment for VL, with drugs like sodium stibogluconate, meglumine antimoniate, paromomycin, miltefosine, and liposomal amphotericin B. However, chemotherapy faces challenges with increasing drug resistance [10]. Mortality in VL is linked to various factors, including jaundice, thrombocytopenia, severe neutropenia, hemorrhage, HIV coinfection, bacterial infection, diarrhea, and others [16].

*Blastocystis* spp. exhibits extensive genetic diversity, with 22 subtypes (STs) identified, and ST1-ST9, as well as ST12, are prevalent in humans [4]. Dogs, cats, pigs, and cattle serve as reservoirs for zoonotic *Blastocystis* subtypes. Transmission routes include fecal-oral (zoonotic, anthroponotic), waterborne, human-to-human, and animal-to-human contact. *Blastocystis* spp. also exhibits morphological diversity, including vacuolar, granular, ameboid, and other forms, with the cyst being considered as the transmission form. There is ongoing debate surrounding *Blastocystis* pathogenicity, but evidence supports its ability to cause infection in animal models and outbreaks among humans. Infections can affect immunocompetent and immunodeficient individuals, including travelers from endemic areas [4]. Clinical manifestations vary globally, with some countries showing higher rates of symptomatic individuals than asymptomatic carriers. Common clinical manifestations include gastrointestinal symptoms, such as diarrhea, abdominal pain, nausea, and vomiting. *Blastocystis* has also been associated with Hashimoto’s thyroiditis, inflammatory bowel disease, skin disorders [4], arthritis, and renal failure [17]. 

The primary diagnostic method for *Blastocystis *is the light microscopic examination of fecal material. Permanent smears are preferred over wet mounts, and trichrome staining is the recommended routine stain. The vacuolar form of *Blastocystis* in fecal samples is approximately 4-15 μm in diameter with 1-4 nuclei, a large central vacuole, a thin cytoplasmic band, and unevenly distributed granules, likely related to cell metabolism or storage. Membrane-enclosed cytoplasmic strands may extend into the central vacuole [18].

*Dientamoeba fragilis* or yeasts are common differential diagnoses for *Blastocystis* spp. [19]. *Dientamoeba fragilis *trophozoites can be detected through the wet mount and trichrome stain in fecal specimens. In formalin-fixed wet mount, *Dientamoeba* trophozoites are 8.5 to 17 μm, round or oval thin-walled cells, with 2 to 6 intracellular darker areas, 0.5 to 1.0 μm in diameter surrounded by a halo [20]. After trichome staining, these trophozoites typically present 1 or 2 nuclei with fragmented nuclear chromatin and lack peripheral chromatin, along with vacuolated cytoplasm containing ingested debris and uniform granules [21]. Yeasts can be differentiated by using GMS or PAS stain.

Although light microscopic examination of fecal material is preferred for identifying* Blastocystis*, they have a low sensitivity with over 50% of infections going undetected. *Blastocystis* can also be diagnosed using cultural, molecular, and immunological methods. Xenic cultures, though more sensitive, are not commonly used in diagnostic labs. Molecular techniques like polymerase chain reaction (PCR) targeting the small subunit ribosomal RNA gene offer higher sensitivity than direct smear and culture but are costlier. In the absence of PCR, using two or more methods is recommended [19]. Antigen antibody-based methods, such as enzyme-linked immunosorbent assay (ELISA), double sandwich ELISA, and indirect immunofluorescence (IFA), can detect *Blastocystis* in stool and serum samples from either symptomatic or asymptomatic patients [22,23]. Metronidazole is the standard treatment for *Blastocystis*. However, a recent trial comparing metronidazole treatment and placebo showed no significant difference in outcomes [24].

A single reported study [25] conducted in India showed that the prevalence of *Blastocystis* in VL was comparable to that in endemic controls. The study was conducted in India, where *Leishmania donovani* is endemic. It is also worth mentioning that the study did not document the clinical characteristics of the participants. It remains unknown whether a similar prevalence of *Blastocystis* in VL occurs in Latin America where *Leishmania chagasi* is endemic.

In our case, the chief initial complaint was several months of recurrent diarrhea, leading to a gastrointestinal infection as the primary initial differential diagnosis. Later on, due to pancytopenia and splenomegaly, malignancy was suspected. Therefore, the presence of *Leishmania* amastigotes in the spleen was unexpected. Although diarrhea can manifest in both visceral leishmaniasis and *Blastocystis* infection, chronic diarrhea is uncommon in VL. The resolution of the patient's diarrhea symptoms after treatment with metronidazole suggests a co-infection of *Leishmania *and *Blastocystis* in this case.

## Conclusions

VL with *Blastocystis* co-infection is uncommon and can present with non-specific clinical manifestation. VL can be fatal if left untreated, therefore, a prompt and accurate diagnosis is critical. Additionally, co-infection can occur, leading to severe manifestations and complexities in treatment. To diagnose VL, a light microscopic examination of the biopsy tissue samples such as spleen, lymph node or bone marrow is recommended, while for *Blastocystis*, a light microscopic examination of fecal material is recommended.
